# Three‐dimensional architecture and moment arms of human rotator cuff muscles in vivo: Interindividual, intermuscular, and intramuscular variations

**DOI:** 10.1111/joa.14050

**Published:** 2024-05-01

**Authors:** Yilan Zhang, Robert D. Herbert, Lynne E. Bilston, Bart Bolsterlee

**Affiliations:** ^1^ Neuroscience Research Australia (NeuRA) Randwick New South Wales Australia; ^2^ Graduate School of Biomedical Engineering University of New South Wales Sydney New South Wales Australia; ^3^ School of Biomedical Sciences University of New South Wales Sydney New South Wales Australia; ^4^ School of Clinical Medicine, Faculty of Medicine & Health University of New South Wales Sydney New South Wales Australia; ^5^ School of Mechanical, Medical and Process Engineering Queensland University of Technology Brisbane Queensland Australia

**Keywords:** diffusion tensor imaging, magnetic resonance imaging, muscle architecture, rotator cuff muscles

## Abstract

The human rotator cuff consists of four muscles, each with a complex, multipennate architecture. Despite the functional and clinical importance, the architecture of the human rotator cuff has yet to be clearly described in humans in vivo. The purpose of this study was to investigate the intramuscular, intermuscular, and interindividual variations in architecture and moment arms of the human rotator cuff. Muscle volumes, fascicle lengths, physiological cross‐sectional areas (PCSAs), pennation angles, and moment arms of all four rotator cuff muscles were measured from mDixon and diffusion tensor imaging (DTI) scans of the right shoulders of 20 young adults. In accordance with the most detailed dissections available to date, we found substantial intramuscular variation in fascicle length (coefficients of variation (CVs) ranged from 26% to 40%) and pennation angles (CVs ranged from 56% to 62%) in all rotator cuff muscles. We also found substantial intermuscular and interindividual variations in muscle volumes, but relatively consistent mean fascicle lengths, pennation angles, and moment arms (CVs for all ≤17%). Moreover, when expressed as a proportion of total rotator cuff muscle volume, the volumes of individual rotator cuff muscles were highly consistent between individuals and sexes (CVs ≤16%), suggesting that rotator cuff muscle volumes scale uniformly, at least in a younger population without musculoskeletal problems. Together, these data indicate limited interindividual and intermuscular variability in architecture, which may simplify scaling routines for musculoskeletal models. However, the substantial intramuscular variation in architecture questions the validity of previously reported mean architectural parameters to adequately describe rotator cuff function.

## Graphical abstract

The human rotator cuff consists of four muscles, each with a complex, multipennate architecture. Despite the functional and clinical importance, the architecture of the human rotator cuff has yet to be clearly described in humans in vivo. In this study, we used advanced magnetic resonance imaging methods to quantify inter‐individual, intermuscular, and intramuscular variations in three‐dimensional architecture of human rotator cuff muscles.

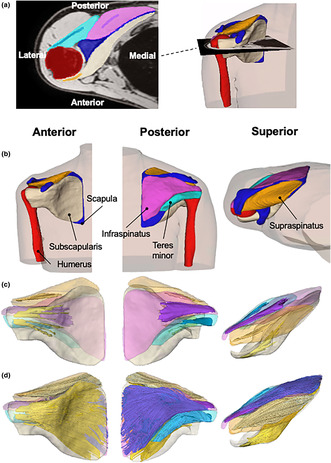

## 1 INTRODUCTION

The force‐generating capacity of a muscle is largely determined by its architecture (muscle volume, fascicle length, pennation angle, and physiological cross‐sectional area (PCSA)) and moment arm (Gans, 1982; Lieber & Fridén, 2000). The muscle fibre length (proportional to maximum muscle excursion) and PCSA (proportional to maximum muscle force) are the primary determinants of the functional capacity of a muscle (Lieber & Fridén, 2000). For a given muscle volume, there is a theoretical and empirical trade‐off between maximizing muscle excursion and maximum shortening velocity (relatively long fibres) and muscle force (relatively large PCSA) (Ward et al., 2009, 2010). Therefore, the architecture of a muscle provides insights into its function. The muscle's moment arm determines how muscle force is converted into joint torque and how changes in muscle length are converted into joint motion (Sherman et al., 2013). Muscle architecture and moment arm measurements are therefore frequently used to infer a muscle's functional capacity and its role in stabilizing and moving joints.

The human rotator cuff consists of four muscles (supraspinatus, subscapularis, infraspinatus, and teres minor) that span the glenohumeral joint. These muscles control shoulder movement and provide dynamic stability at the joint. Rotator cuff tears account for up to 50% of major shoulder injuries and there is a high rate of retear (34%–65%; Gazielly et al., 1994; Gerber et al., 2000). Moreover, rotator cuff tears are usually accompanied by changes in muscle architecture which reverse slowly or may be irreversible. The inability to predict muscle function at the time of surgery could contribute to the high rate of failure (Wellmann, 2016).

Despite its functional and pathophysiological importance, the architecture of rotator cuff muscles has not yet been clearly and comprehensively described. Rotator cuff muscles are multipennate muscles – their fascicle lengths and pennation angles vary between anatomically distinct regions (Kim et al., 2007). In a cadaveric study using detailed dissection techniques, Ward et al. (2006) found differences in fibre length between regions of the same muscle of around 20%, with the largest difference (27%) found between the superior and inferior regions of the subscapularis. Similarly, Roh et al. (2000) found significant regional differences with larger muscle volume (200%), mean fibre length (27%), mean pennation angle (40%), and PCSA (125%) in the anterior region compared to the posterior region of supraspinatus muscles of 25 embalmed cadavers. Regionalization may allow a variety of force vectors to exist within a single muscle (Herring et al., 1979), and challenges the common assumption that a muscle's function can be described accurately by its mean architectural parameters.

Rotator cuff muscle architecture differs not only within muscles, but also between muscles, allowing them to jointly generate the forces needed to move the shoulder and prevent dislocation of the glenohumeral joint (Keating et al., 1993). From dissections of cadaveric rotator cuff muscles, Keating et al. (1993), Ward et al. (2006), and Mathewson et al. (2014) consistently found the subscapularis to have the largest PCSA, followed by the infraspinatus, supraspinatus, and teres minor. These studies also reported considerable interindividual variation in architecture, although the variability was not quantified in the study by Keating et al. (1993) potentially due to the small number of subjects included (*n* = 5). Previous cadaveric studies have quantified and reported intramuscular (Langenderfer et al., 2006) and intermuscular variations (Hik & Ackland, 2019) in moment arms of rotator cuff muscles during different shoulder movements. Furthermore, several studies have reported sex‐related differences in muscle size (Kälin et al., 2018), function (Motabar & Nimbarte, 2021) and prevalence of rotator cuff tears (Abate et al., 2014).

Computational modelling is commonly used to investigate musculoskeletal biomechanics because muscle forces are usually difficult or impossible to measure in vivo. Computational modelling of rotator cuff muscles is also used clinically, for example, to predict the effect of rotator cuff tears on muscle and glenohumeral joint contact forces (Khandare et al., 2022; Vidt et al., 2018) and to inform implant design for shoulder arthroplasty (Büchler & Farron, 2004). Despite the intrinsic complexity and interindividual variation of shoulder muscle architecture, most computational models are generic models based on measurements obtained from one cadaver specimen, sometimes scaled to an individual's anthropometry. The inability to fully capture interindividual differences in joint anatomy and muscle architecture with scaled‐generic models may lead to inaccurate model predictions. Sensitivity analyses have quantified the inaccuracies in muscle and joint force predictions related to inaccuracies in muscle architecture parameters (Bates & Falkingham, 2018; Broyde et al., 2021; Charles et al., 2022; Gröning et al., 2013; Kramer et al., 2022; Persad et al., 2023).

Clearly, there is need for accurate subject‐specific assessment of rotator cuff muscle architecture for both clinical and computational studies. B‐mode ultrasound imaging is often used to measure architecture of human muscles in vivo (Franchi et al., 2018), including the supraspinatus muscle (Kim et al., 2010, 2013). However, conventional ultrasound imaging has significant limitations. First, architecture measurements from ultrasound are prone to projection and extrapolation errors because the 3D trajectories of fibres cannot be captured on a single 2D image (Bolsterlee et al., 2016). Moreover, changes in probe orientation, contraction‐related changes in muscle orientation, or pressure applied by the probe on muscle can induce errors (Van Hooren et al., 2020). Another limitation is that ultrasound waves cannot penetrate bones, so large parts of the subscapularis cannot be imaged (Zhang et al., 2023). As a result, most data on rotator cuff muscle architecture have been obtained from measurements made on cadavers, typically from elderly people (Keating et al., 1993; Mathewson et al., 2014; Ward et al., 2006). These measurements do not provide insights into the variability in rotator cuff architecture in living humans.

In recent years, diffusion tensor imaging (DTI) has emerged as an alternative method to study skeletal muscle architecture in vivo. DTI is an MRI‐based technique that exploits the principle of restricted water molecule diffusion in muscle fibres to reconstruct muscle architecture in 3D space (Damon et al., 2017). Muscle architectural parameters, such as fascicle length, pennation angle, and PCSA, can then be measured from DTI‐based reconstructions with good reliability (Bolsterlee et al., 2019), including in complex, regionalised muscles such as the human soleus (Bolsterlee et al. (2018)). Aeles et al. (2022) used DTI methods to demonstrate substantial regional variation in fibre lengths in both the medial and lateral gastrocnemius, and interindividual variation in regional fibre lengths. Using similar methods, another study reported regional variations in both fibre lengths and pennation angles within human medial gastrocnemius muscles (Takahashi et al., 2022).

We have recently used DTI and anatomically constrained tractography to reconstruct and quantify the human subscapularis muscle architecture in vivo (Zhang et al., 2023). Here, we extend these techniques to all rotator cuff muscles, aiming to (1) provide comprehensive data on rotator cuff muscle architecture and moment arms and (2) investigate the intramuscular, intermuscular, and interindividual variations in muscle architecture and moment arms of rotator cuff muscles in healthy young adults.

## 2 METHODS

### 2.1 Participants

We used data collected previously to develop methods to reconstruct the human subscapularis architecture (Zhang et al., 2023). Study procedures were approved by the UNSW Human Research Ethics Committee (HREC approval HC200971). Twenty adults (11 males and 9 females; age 28 ± 6 years; height 171 ± 8 cm; weight 64 ± 11 kg, values are mean ± standard deviation) with no symptoms or recent history of shoulder pathology participated in this study. All provided written informed consent prior to participation.

### 2.2 MRI acquisitions

The participants' right shoulders were examined in a 3 T MRI scanner (Philips Ingenia CX, Philips Healthcare, Best, The Netherlands) using a 16‐channel anterior body coil and a 16‐channel posterior coil integrated in the scanner bed. Participants lay in a head‐first position with the arms resting alongside the torso and the right palm facing up. Prior to the scan, the participant was asked to slide to the left side of the MRI bed to place the right shoulder as close as possible to the centre of the scanner bore.

The MRI examination consisted of an mDixon scan obtained in the sagittal oblique plane for anatomical reference and two diffusion‐weighted scans covering the proximal and distal rotator cuff musculature, respectively, for muscle architecture reconstruction. Having derived the diffusion tensor from diffusion‐weighted image data in our analysis, we will henceforth refer to these scans as DTI scans. The scan parameters for mDixon images were as follows: two‐point 3D T1‐Fast Field Echo (FFE) sequence, TR/TE_1_/TE_2_ 6.0/3.5/4.6 msec, field of view (FOV) 240 mm with voxel size 1 × 1 × 2 mm, acquisition matrix 240 × 198 (reconstructed to 320 × 264), 210 slices, number of signal averages 2, and scan time 6 min. The DTI parameters were as follows: single‐shot Echo‐Planar Imaging (EPI), TR/TE 3000/46 msec, FOV 190 mm with voxel size 2.5 × 2.5 × 5 mm, slice gap 0.5 mm, acquisition matrix 76 × 76 (reconstructed to 240 × 240), 24 slices, 12 diffusion encoding directions on a hemisphere, *b* = 0 and 500 s/mm^2^, number of signal averages 9 and total scan time 13 min for both scans combined. Two sets of *b* = 0 images with opposite polarity of the phase‐encoding direction were acquired to enable correction for susceptibility‐induced geometric and intensity distortions (Andersson et al., 2003; Smith et al., 2004).

### 2.3 Image segmentation

**FIGURE 1:**
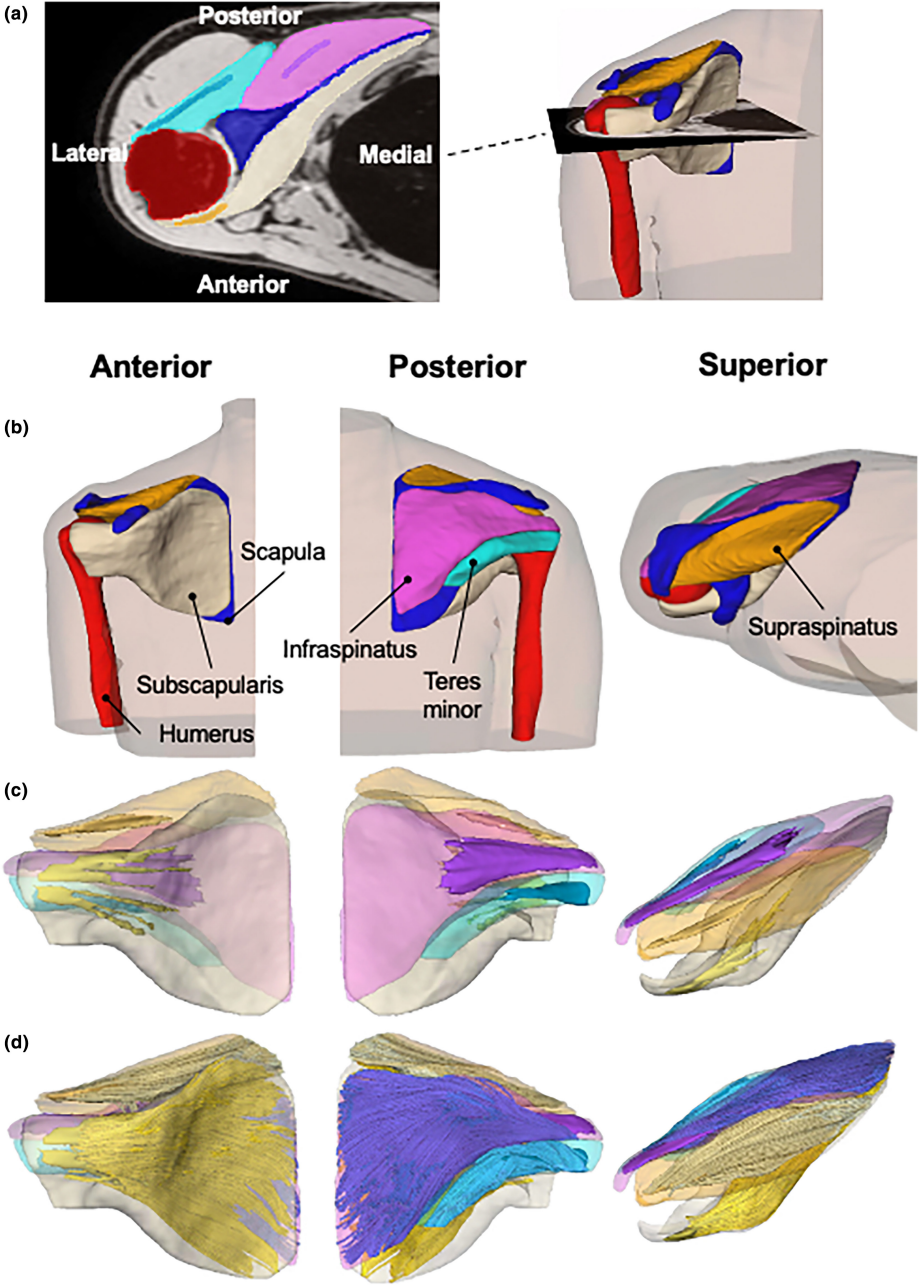
Example of a reconstruction of the 3D muscle architecture of human rotator cuff muscles from anatomical and DTI scans. (a) Transverse slice of the mDixon water image approximately midway through the glenohumeral joint. Bones, muscles, and internal aponeuroses are shaded (red, humerus; blue, scapula; beige, subscapularis; pink, infraspinatus; cyan, teres minor; orange, supraspinatus). Aponeuroses appear as darker lines within the belly of the muscle. (b) 3D surface models of rotator cuff muscles and (c) internal aponeuroses. (d) 3D fascicle reconstructions of rotator cuff muscles made with anatomically constrained DTI tractography.

Muscle and bone segmentation was carried out semi‐automatically on mDixon scans (Figure 1a). A deep learning model (nnU‐net; Isensee et al., 2021) was trained using 12 manually segmented mDixon shoulder scans obtained previously in our laboratory using the same image protocol, and used to predict the segmentation of rotator cuff muscles and bones (humerus, scapula and clavicle) in all 20 scans used in this study. All predicted segmentations were visually inspected and manually corrected where necessary by one experimenter (Y.Z.) using ITK‐SNAP (Yushkevich et al., 2006). In four of the twenty scans, it was not possible to accurately locate the boundary between the infraspinatus and teres minor, so these muscles were grouped together.

Using the mDixon water images, the internal aponeurosis of each muscle was segmented by manually selecting low‐intensity voxels within the muscle. Collagen‐rich tissues such as aponeuroses have very low transverse relaxation times and therefore have low signal intensities on most MR scans (Bird et al., 2019).

From the segmentations, 3D surface models were generated for all muscles (Figure 1b) and internal aponeuroses (Figure 1c) by applying the marching cubes algorithm (Lorensen & Cline, 1987) in MRtrix (MRtrix3; Tournier et al., 2019). The volume of individual muscles was determined from the volume enclosed by the surface model.

### 2.4 DTI post‐processing

DTI scans were post‐processed in MRtrix to correct image artefacts and improve signal‐to‐noise ratio. A Marchenko–Pastur principal component analysis filter (Veraart, Fieremans, & Novikov, 2016; Veraart, Novikov, et al., 2016) was applied to DTI data to reduce image noise. The denoised DTI scans were then corrected for eddy current‐ and possible motion‐induced distortions using functions TOPUP and EDDY built into FSL (Andersson & Sotiropoulos, 2016). DTI scans were linearly upsampled to the mDixon image grid (resolution, image size, and coordinate space) using the mrgrid function from MRtrix (Tournier et al., 2019), after which the DTI scans were stitched together in MATLAB (version: R2021a) to generate a single DTI image which covered the entire rotator cuff. Values in overlapping regions between scans were averaged to ensure a smooth transition between stacks. To correct for small misalignments between mDixon and DTI scans within subjects, the mDixon image was rigidly registered to the b_0_ image of the stitched DTI scan using the FLIRT function from FSL (Jenkinson et al., 2002; Jenkinson & Smith, 2001) with six degrees of freedom. The alignment after image registration was visually inspected and confirmed by overlaying the registered mDixon image on the stitched DTI scan.

### 2.5 Muscle architecture measurements

DTI tractography was performed using a deterministic fibre tracking algorithm (Basser et al., 2000) and anatomically constrained tractography (Smith et al., 2012), built into MRtrix (Tournier et al., 2019). Detailed descriptions of fibre tracking procedures can be found in our previous work (Zhang et al., 2023). In brief, 3000 tracts were generated in each muscle (settings: integration step size = 1.0 mm; 0.1 ≤ fractional anisotropy ≤ 0.5; maximum turning angle between successive steps =15°; 25 mm ≤ tract length ≤ 200 mm). Using the anatomically constrained tractography framework built into MRtrix (Smith et al., 2012), each tract connected the boundary of the aponeurosis with the muscle surface. All fascicle reconstructions were visually inspected in MATLAB and MRtrix for plausibility and to confirm that muscle fascicles terminate on the surface of the aponeuroses (Figure 1d).

For each muscle, 3D architectural measurements were obtained from the fascicle reconstructions. Mean fascicle length was defined as the average length of all reconstructed fibre tracts. Pennation angle was determined as the average angle between fascicles and all faces of the aponeurosis surface model within 1.5 mm of the end point of each fascicle. PCSA, which equals the sum of the cross‐sectional areas of all fibres within a muscle, was calculated by dividing muscle volume by mean fascicle length, which assumes that the muscle was composed only of muscle fibres (D'Souza et al., 2019).

### 2.6 Muscle moment arms

**FIGURE 2:**
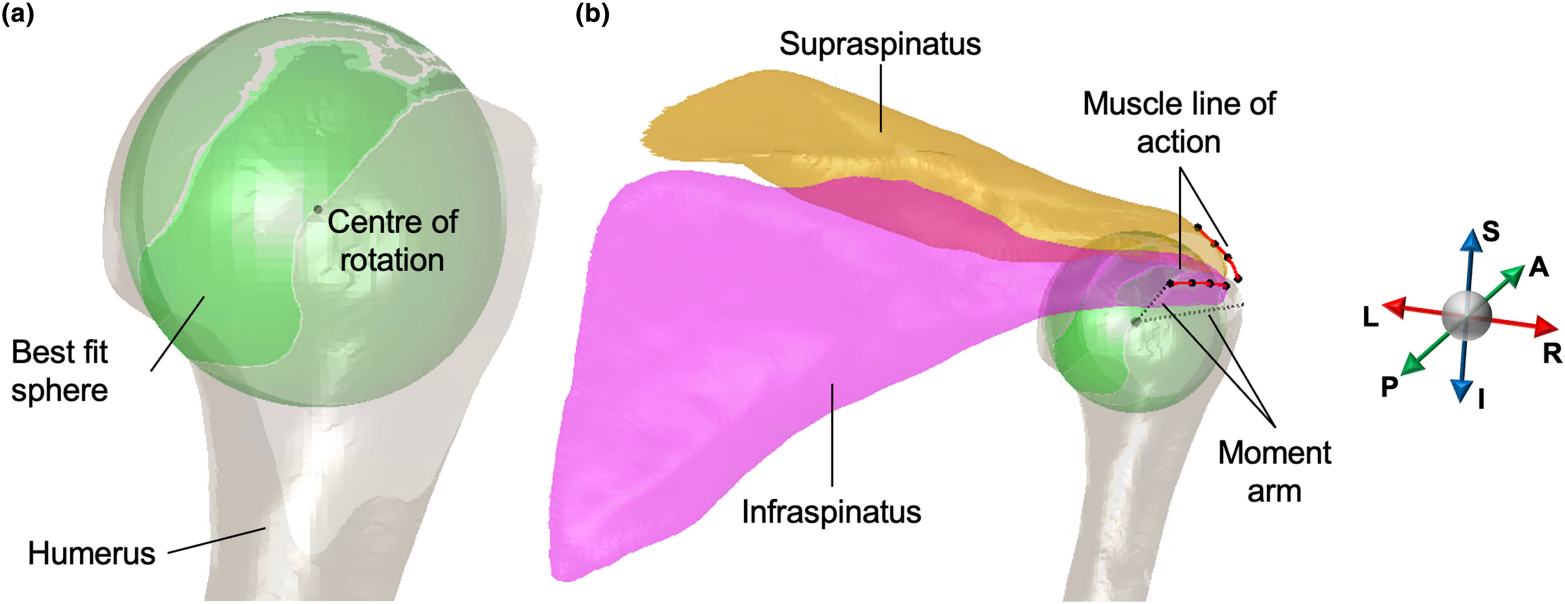
Posterior view of the shoulder showing the procedure used to measure glenohumeral moment arms of the infraspinatus (pink) and supraspinatus (orange) muscles. (a) A sphere (green) was fitted to the articular surface of the humeral head, the centre of which approximated the glenohumeral centre of rotation. (b) The moment arm of each muscle was calculated as the shortest perpendicular distance between the muscle line of action and the centre of rotation.

Glenohumeral moment arms of all rotator cuff muscles were estimated by a 3D geometric method (Meskers et al., 1997; Figure 2). The glenohumeral joint centre of rotation was estimated by finding the centre of the best fit sphere to the articular surface of the reconstructed humeral head (Figure 2a). The muscle line of action was reconstructed from a third‐order polynomial curve fitted through a cluster of four landmarks manually placed on the outer surface of each rotator cuff tendon. The first landmark was set at the midpoint of the muscle's insertion on the humeral head. Subsequently, the positioning of additional landmarks was guided by the predominant orientation of the internal fibre tracts, serving to delineate the muscle line of action. The moment arm was then defined as the shortest perpendicular distance between the muscle line of action and the centre of rotation (Figure 2b).

### 2.7 Reliability

The intrarater reliability of muscle volume and moment arm measurements was determined by repeating the mDixon scans using the same image protocol, segmentation, and moment arm calculation method on 10 of the 20 participants. The interval between repeated scans was approximately one year. The same experimenter (Y.Z.) performed the segmentation and moment arm measurements using de‐identified and randomly ordered mDixon data.

### 2.8 Statistical analysis

Intramuscular variation in fascicle length and pennation angle was assessed for each muscle of each participant by calculating the intramuscular coefficient of variation (CV), calculated as the ratio of the standard deviation and mean of fascicle lengths and pennation angles across 3000 reconstructed fibre tracts per muscle. Intermuscular variation was assessed by comparing the mean fascicle lengths and pennation angles between muscles. Interindividual variation was assessed as the CV calculated from the mean and standard deviations across participants of the mean architectural parameter of each muscle. An unpaired t‐test was carried out to compare muscle architectural parameters and moment arms between males and females.

The intrarater reliability of muscle volume and moment arm measurements was assessed by calculating the absolute‐agreement intraclass correlation coefficient ([ICC (2,1)] with 95% confidence interval (CI); Shrout & Fleiss, 1979) and mean absolute difference between paired measurements. All statistical analyses were conducted using SPSS Version 27.0 (Armonk, NY: IBM Corp).

## 3 RESULTS

Architectural parameters were successfully measured on all muscles from all participants. The data including MRI and DTI scans, segmentations, 3D surface models and fibre tracts will be made available for research purposes on request.

### 3.1 Intrarater reliability

The intrarater reliability for muscle volume and moment arm measurements was good to excellent (ICCs 0.86 to 0.99; Table 1), with the lowest ICC found for the teres minor.

**TABLE 1:** Muscle volume and moment arm data from two measurements performed on the same group of subjects (means ± SDs) and the intrarater reliability, expressed as intraclass correlation coefficients (ICC) and 95% confidence intervals (CI).

Measure		Measurement 1 (*n* = 10)	Measurement 2 (*n* = 10)	ICC	95% CI	Mean absolute difference (%)
Muscle volume (cm^3^)	Supraspinatus	48 ± 15	47 ± 15	0.99	0.97–1.00	3.4
	Subscapularis	135 ± 43	135 ± 45	0.99	0.96–1.00	3.6
	Infraspinatus	111 ± 41	110 ± 40	0.99	0.97–1.00	3.6
	Teres minor	25 ± 9	21 ± 9	0.89	0.89–0.98	17.5
Moment arm (mm)	Supraspinatus	24.6 ± 0.9	24.7 ± 0.7	0.88	0.60–0.97	1.3
	Subscapularis	23.2 ± 1.5	23.4 ± 1.5	0.97	0.88–0.99	1.4
	Infraspinatus	24.0 ± 1.7	24.1 ± 1.6	0.97	0.90–0.99	1.3
	Teres minor	22.9 ± 1.5	22.9 ± 1.4	0.86	0.57–0.96	2.8

### 3.2 Muscle architecture and moment arms

Muscle architectural properties and moment arms for all muscles and participants are summarised in Table 2. In four of the 20 scans, it was necessary to group the infraspinatus and teres minor muscles together; data for these muscles from those four subjects are reported separately. Data for individual participants can be found in Table S1 in the Supplementary Material.

**TABLE 2:** Muscle architecture and moment arm measurements of rotator cuff muscles.

	Muscle volume (cm^3^)	Relative volume (% of total)	PCSA (cm^2^)	Fascicle length (mm)	Pennation angle (°)	Moment arm (mm)
Supraspinatus (*n* = 20)	47 ± 12 (26)	15 ± 2 (12)	8 ± 2 (30)	59.8 ± 7.2 (12)	17 ± 3 (15)	24.2 ± 1.6 (7)
Subscapularis (*n* = 20)	138 ± 42 (30)	43 ± 2 (6)	23 ± 7 (31)	61.8 ± 8.7 (14)	21 ± 2 (9)	23.3 ± 1.6 (7)
Infraspinatus (*n* = 16)	113 ± 36 (32)	34 ± 2 (6)	17 ± 5 (29)	69.7 ± 11.7 (17)	20 ± 2 (11)	24.2 ± 2.0 (8)
Teres minor (*n* = 16)	28 ± 10 (36)	8 ± 1 (16)	6 ± 2 (37)	41.9 ± 5.2 (13)	22 ± 3 (12)	22.9 ± 1.4 (6)
Infraspinatus + teres minor (*n* = 4)	120 ± 31 (26)	42 ± 17 (3)	19 ± 5 (24)	62.6 ± 2.6 (4)	21 ± 2 (8)	24.7 ± 1.1 (5)

*Note*: Values are means ± SDs (% CV) across participants.

#### 3.2.1 Intramuscular variation

**FIGURE 3:**
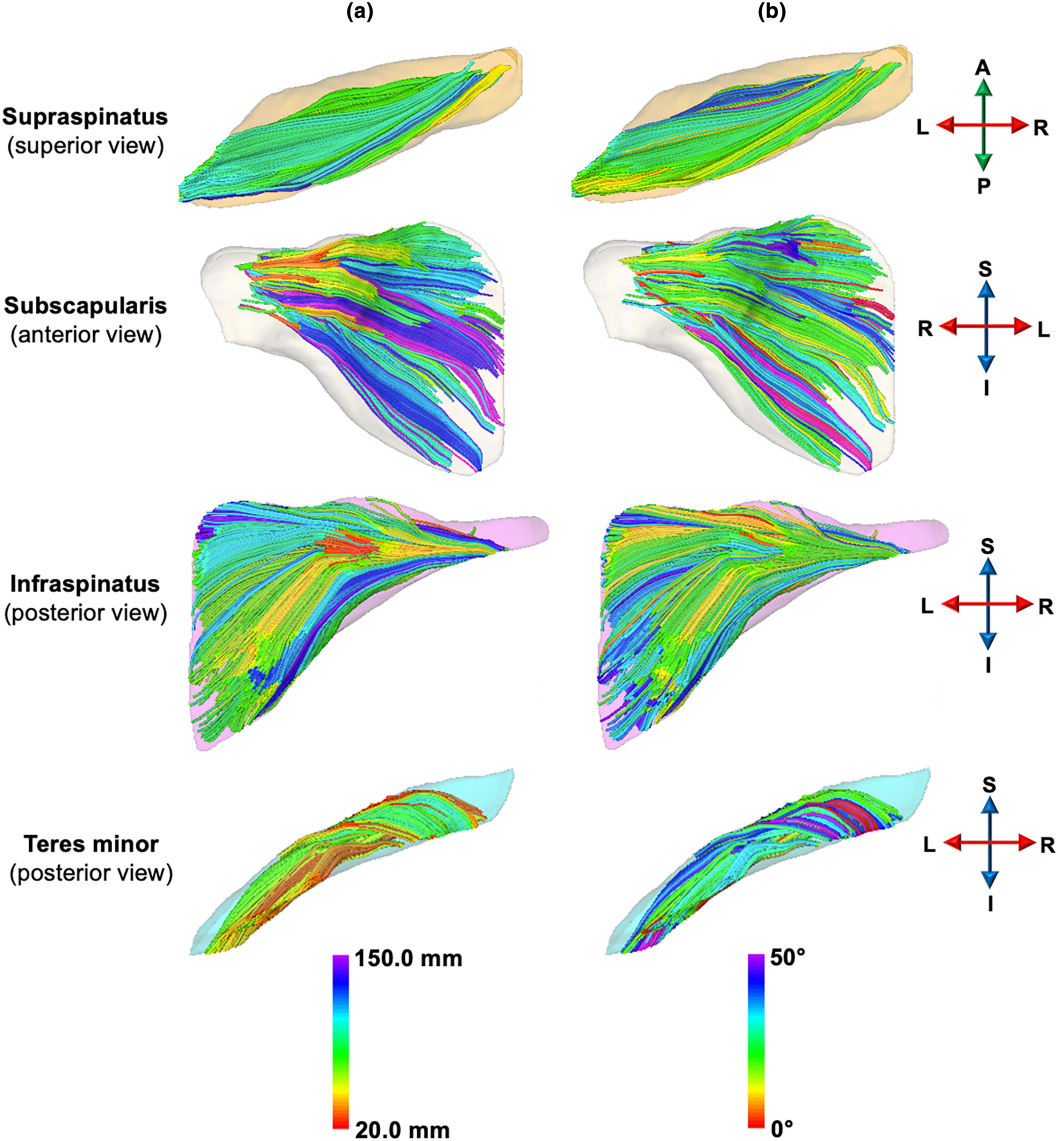
Representative 3D fascicle reconstructions of rotator cuff muscles of one participant. Reconstructed fibre tracts are colour‐coded by their (a) length and (b) pennation angle.

Fascicle reconstructions showed a dense distribution of fibres that had varying lengths and pennation angles within muscles (Figure 3). There was substantial intramuscular variability in pennation angle (CV = 58% ± 2%, averaged across all muscles and participants; Table S1). Muscle fascicle length also varied within muscles (Table S1), with CVs ranging from 26% ± 5% (teres minor; averaged across all participants) to 40% ± 8% (subscapularis; averaged across all participants).

#### 3.2.2 Intermuscular variation

There was a nearly fivefold variation in mean muscle volume between muscles (Table 2). In all participants, the subscapularis muscle was the largest muscle with a mean volume of 138 cm^3^ (43% of the total rotator cuff muscle volume). The next largest muscles were, in decreasing order of size, infraspinatus (34%), supraspinatus (15%), and teres minor (8%).

Intermuscular variation in PCSAs was similar to that in muscle volumes. The subscapularis had the largest PCSA (23 cm^2^), followed by the infraspinatus (17 cm^2^), supraspinatus (8 cm^2^), and teres minor (6 cm^2^).

**FIGURE 4:**
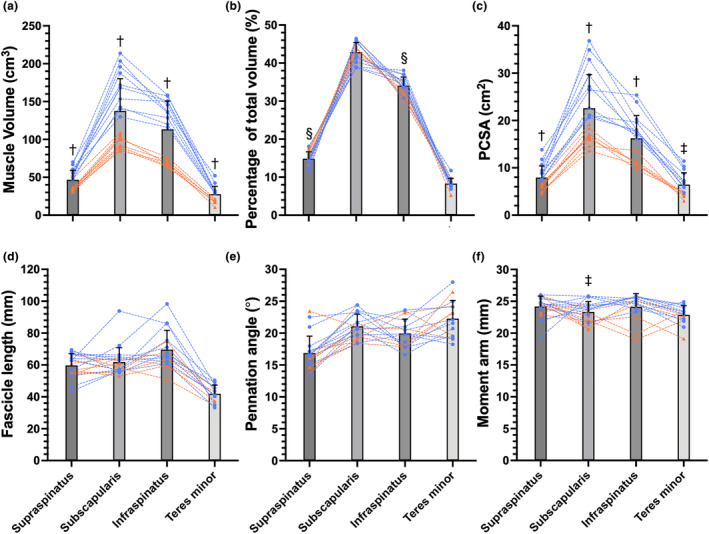
Muscle architecture and moment arm measurements of supraspinatus (*n* = 20), subscapularis (*n* = 20), infraspinatus (*n* = 16) and teres minor (*n* = 16), presented as (a) muscle volume, (b) volume percentage, (c) PCSA, (d) fascicle length, (e) pennation angle, and (f) moment arm. Dashed lines connect the measurements made on the same subject (blue: male; orange: female). The bars indicate means ± SDs across participants. Symbols above the bars indicate statistically significant differences between males and females (^†^ *p* < 0.001; ^‡^ *p* < 0.01; ^§^ *p* < 0.05).

There was considerably less intermuscular variation in fascicle lengths, pennation angles and moment arms. Mean fascicle lengths were shortest in the teres minor (mean 41.9 mm) and longest in the infraspinatus (mean 69.7 mm). Mean pennation angles (ranging from 17 to 22°) and moment arms (ranging from 22.9 to 24.2 mm) varied little between muscles (Table 2 and Figure 4).

#### 3.2.3 Interindividual variation

The interindividual variability was greatest in muscle volumes (CVs ranged from 26% to 36%) and PCSAs (CVs ranged from 24% to 37%). There was relatively little interindividual variability in relative volumes, fascicle lengths, pennation angles, and moment arms (CVs for all ≤17%; Table 2).

The volumes and PCSAs of all rotator cuff muscles were, on average, greater in males than in females (*p* < 0.001, Figure 4a,c) by 45 cm^3^ (83%) and 7 cm^2^ (70%; means are across all muscles and participants). Sex differences remained but were less obvious when muscle volume was expressed as a percentage of total rotator cuff muscle volume (*p* < 0.05 for supraspinatus and infraspinatus; *p* ≥ 0.05 for the other two muscles; Figure 4b). Compared with females, relative volumes of the supraspinatus and subscapularis were 2% and 1% larger in men, while the infraspinatus and teres minor were 2% and <1% smaller. There were no statistically significant differences in mean fascicle length (*p* ≥ 0.05), pennation angle (*p* ≥ 0.05), or moment arm (*p* ≥ 0.01 for subscapularis; *p* ≥ 0.05 for the other three muscles) between males and females.

## 4 DISCUSSION

This study quantified the 3D architecture and moment arms of rotator cuff muscles in an adult population without symptoms or recent history of shoulder pathology, and compared architectural measurements within muscles, between muscles, and between individuals. We found significant intramuscular variation in both fascicle length and pennation angle, as well as substantial intermuscular and interindividual variations in muscle volumes. In contrast, mean fascicle lengths, mean pennation angles, moment arms, and relative muscle volumes were relatively consistent between individuals and sexes.

In the current manuscript, we report the mean fascicle lengths, a common practice in the majority of studies in the field. Charles et al. (2022) suggested that, particularly for muscles with irregular and complex designs, fascicle lengths may not follow normal distributions and could be better summarised by median values. In our data, mean fascicle lengths were slightly larger than median fascicle lengths, differing by an average of 1.9 mm across all muscles and participants (Table S2). Therefore, we have decided to report the mean fascicle length in the text.

### 4.1 Intramuscular variation

Our data showed that both fascicle length and pennation angle varied substantially within muscles, with the degree of variation relatively consistent between individuals. The intramuscular variations reported in this study are qualitatively consistent with previous cadaver studies which reported regional differences in fascicle length and pennation angle within rotator cuff muscles (Bacle et al., 2017; Kim et al., 2007; Ward et al., 2006). Intramuscular variations in muscle architecture likely lead to their intramuscular functional heterogeneity. This is supported by previous studies which revealed specific functions for different subregions of the supraspinatus (Gates et al., 2010) and infraspinatus (Kuwahara et al., 2017). Furthermore, such intramuscular or regional heterogeneity suggests that whole‐muscle force is the result of a complex interplay between muscle fascicle forces with a range of directions and magnitudes within a single muscle. This intricate dynamic could have significant implications for understanding how specific muscle regions contribute to whole muscle function and, ultimately, to particular aspects of joint motion or stability. A previous study found that individual fascicles could reach their optimal lengths at different whole‐muscle lengths (Higham & Biewener, 2011), enabling effective muscle function over a wide range of muscle lengths. Additionally, the selective recruitment of distinct rotator cuff muscle regions, each characterised by unique architectural designs, likely enables the generation of different torques about a joint (Carrasco et al., 1999), allowing the execution of various joint motions and fine control over joint mechanics. The precise interplay between functional heterogeneity and variations in fascicle lengths and pennation angles within muscles remains unclear, but could be studied using, for example, finite element models built on the geometrical data provided in this study.

In this study, we have likely overestimated the intramuscular variation in architecture, because CV values reported here are a combination of true architectural variation and variation due to measurement error. In deterministic fibre tracking algorithms, fibre tracts are propagated by following the principal eigenvectors of diffusion tensors throughout a volume. Deviations of fibre tracts from actual muscle fibre trajectories can accumulate (Schenk et al., 2013), generating fibre tracts whose lengths and pennation angles are smaller or larger than true values, increasing variation and CVs. It is difficult to determine from currently available data how much of the measured CVs is due to measurement error. Most cadaveric studies, even those using 3D digitisation to reconstruct the 3D trajectories of many fascicles, have reported only means and SDs of fibre lengths and pennation angles in muscle subregions, averaged across subjects (Kim et al., 2007, 2010). Quantitative comparison of the variability of our data obtained at the level of individual fascicles with data from previous studies obtained at the whole muscle level is therefore generally not possible. An exception is for the data reported by Lee et al. (2015), who digitised the trajectories of 1750 fascicles of a supraspinatus muscle and reported the mean and standard deviation of their pennation angles. Our in vivo measurements agree well with their data for the supraspinatus muscle (Lee et al: mean 3D pennation angle of 17 ± 10°, intramuscular CV = 58%; our study: 17 ± 10°, average intramuscular CV of 62% ± 9%). Lee and colleagues did not report fascicle lengths of the supraspinatus, or architectural data on other rotator cuff muscles. In the future, reporting fascicle‐level data from studies of 3D digitisations of rotator cuff muscles would help determine the accuracy of fascicle‐level data measured with DTI.

### 4.2 Intermuscular variation

The muscle architecture and moment arm data presented here are mostly similar to those reported in cadaver studies (Mathewson et al., 2014; Ward et al., 2006) and MRI‐based studies conducted in vivo (Juul‐Kristensen et al., 2000; Table S3), demonstrating the plausibility of our measurements. However, pennation angles reported in this study differed substantially from those reported in cadaver studies (Table S3). Age‐related decreases in pennation angle, as has been described previously (Narici et al., 2003), is one explanation for the smaller pennation angles reported in cadaver studies (Ward et al. (2006): mean 89 years; Juul‐Kristensen et al. (2000): mean 79 years; Mathewson et al. (2014): age not reported, our study: mean 28 years). Additionally, our method involved measurements of angles in 3D between fascicles and the aponeurosis, as opposed to the single‐line measurements of fascicle‐to‐tendon axis in cadaver studies, so methodological differences may contribute to the difference too.

The relatively small intermuscular variations in mean fascicle lengths, pennation angles, and moment arms are consistent with previous studies (Juul‐Kristensen et al., 2000; Mathewson et al., 2014; Ward et al., 2006). A possible exception is the teres minor, which had the shortest mean fascicle length (41.9 mm) of the four muscles examined. The value reported here was 19.0 mm (31%) and 30.6 mm (42%) smaller than the means reported by Ward et al. (2006) and Mathewson et al. (2014), respectively. The relatively low reliability of teres minor segmentations (Table 2) may partially explain the shorter fascicle lengths in this study, but other inaccuracies in DTI fibre tracking may have contributed as well.

**FIGURE 5:**
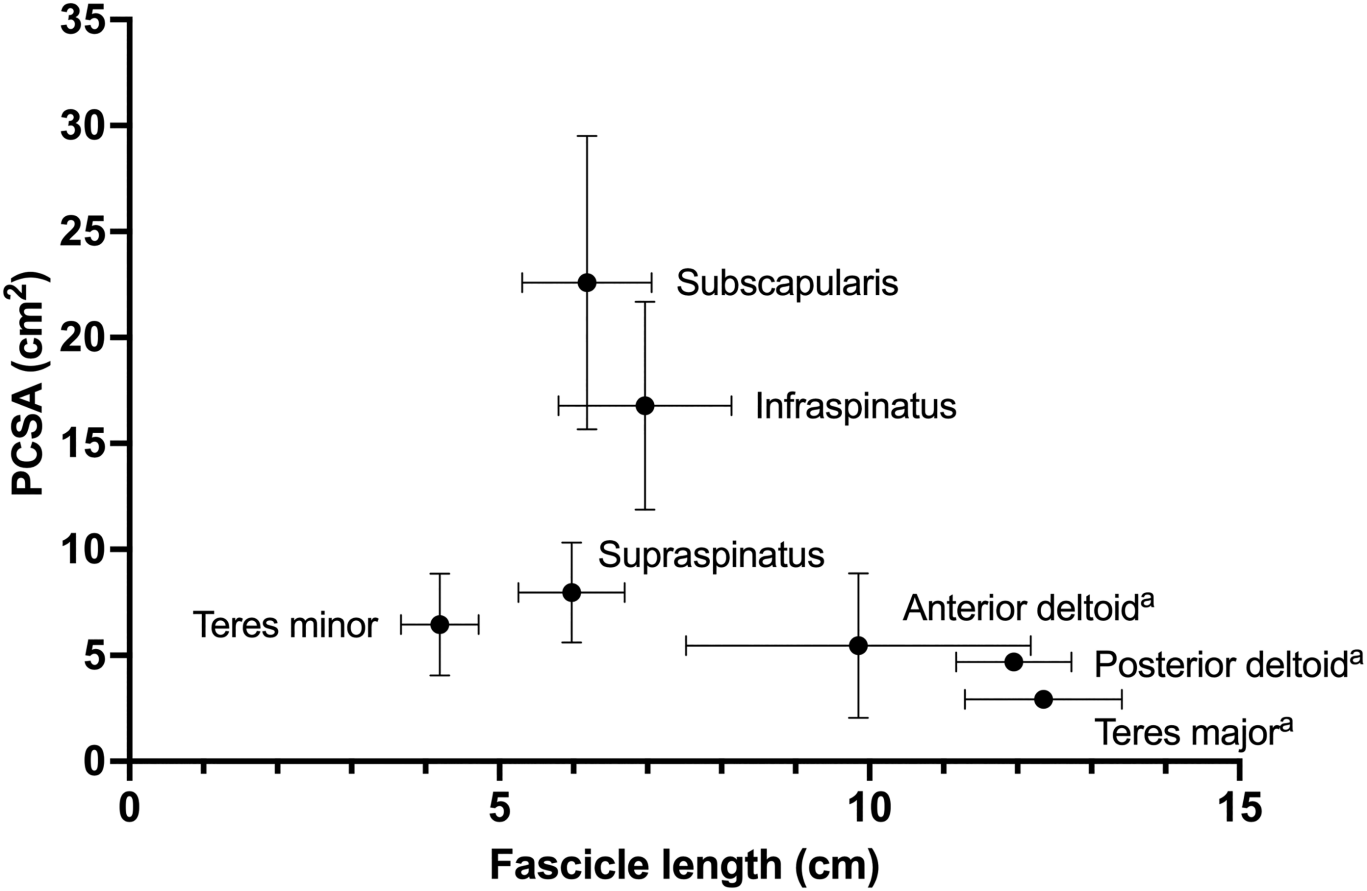
Scatter plot of fascicle length and physiological cross‐sectional area (PCSA) of rotator cuff muscles, deltoid and teres major. ^a^Data from Langenderfer et al. (2004).

Comparison between muscles of location on a fascicle length vs PCSA plot (Figure 5) confirms the predominantly stabilising function of the rotator cuff. Compared to the rotator cuff muscles, other muscles crossing the glenohumeral joint such as the deltoid and teres major muscles have longer fascicle lengths and smaller PCSAs (Langenderfer et al., 2004), and can therefore produce intermediate forces over a relatively large range (Hess, 2000). The rotator cuff muscles, on the other hand, generally have shorter fascicles but relatively large PCSAs, suggesting their primary function as force generators across a relatively narrow range of lengths. Their stabilising function is further confirmed by the rotator cuffs' relatively small moment arms compared to the deltoid (Hik & Ackland, 2019).

Interestingly, the PCSA of the subscapularis (23 cm^2^) equalled the sum of PCSAs of infraspinatus (17 cm^2^) and teres minor (6 cm^2^). When co‐activated, these muscles can generate torques that balance each other in the transverse plane to stabilise the humeral head during glenohumeral motion (Hik & Ackland, 2019; Hughes, 1996). Although the supraspinatus only contributes about 15% of total rotator cuff muscle PCSA, so that it potentially generates smaller torques at the shoulder than the subscapularis and infraspinatus, it has a much higher incidence of injury (Ferrari et al., 2002). The active involvement of the supraspinatus in particular shoulder movements, especially early coronal plane abduction, scaption and flexion, could explain the high injury prevalence (Hik & Ackland, 2019). This distribution of force‐generation capacity among the rotator cuff muscles illustrates a sophisticated balance between stability and mobility in shoulder function, highlighting the intricate interplay of anatomical structure and biomechanical performance.

### 4.3 Interindividual variation

There was little variation in fascicle lengths, pennation angles and moment arms between individuals and between sexes. In contrast, the variations in muscle volumes and PCSAs were large. It is not surprising that males displayed greater muscle volumes and PCSAs for all rotator cuff muscles, as there is a wealth of literature reporting sex differences in muscle mass, especially in the upper limb (Janssen et al., 2000; Miller et al., 1993). However, relative muscle volumes of each muscle, calculated as the percentage of total rotator cuff muscle volume, were very consistent between individuals and sexes. This is also evident in data reported in cadaveric studies (Mathewson et al., 2014; Ward et al., 2006). The implication is that rotator cuff muscles grow in synchrony with each other to maintain a balance of power across the glenohumeral joint. The consistency in relative muscle volumes implies that by measuring the muscle volume of one rotator cuff muscle, volumes of the other muscles could be predicted accurately, at least in healthy populations. This finding also has potential implications for scaling of musculoskeletal models. Bolsterlee et al. (2015) compared the effects of performing uniform scaling and muscle‐specific scaling of PCSA on predictions of a musculoskeletal model of the upper extremity, finding minimal difference between scaling methods in predictions of maximum strength. The use of uniform PCSA scaling was therefore recommended due to its simplicity. That recommendation is strengthened by our finding that there is little between‐person variation in the relative volumes of the rotator cuff. Our data may help modellers determine the degree to which models should be individualised, balancing the time and effort required to generate subject‐specific models against the expected improvements in model predictions, and may assist modellers in identifying which model parameters, if any, need to be individualised.

### 4.4 Limitations

The study has a number of limitations. First, we reported measured fascicle lengths instead of optimal fascicle lengths normalised to sarcomere lengths, because optimal fascicle lengths are not measurable from DTI. As our measured PCSAs were not calculated at optimal fascicle lengths, they cannot be used to infer maximum isometric tension without additional data or assumptions. Second, participants were placed in a position of slight external rotation (<20°) in order to fit in the MRI scanner. The exact degree of rotation was not strictly controlled or measured and could vary slightly between participants due to individual size differences. Unfortunately, the humeral condyles were outside the field of view of the scan, so we could not determine the joint position retrospectively. However, we do not think that variations in glenohumeral joint angles have had a large influence on moment arm measurements. A study by Hik and Ackland (2019) found that variations in rotator cuff muscle moment arms within the 0–20° rotation range are typically less than 5 mm. We estimate that between‐participant variation in joint position in our study was <10°, so our measurements are likely reflective of moment arms within the anatomically neutral position. A third limitation is that we did not quantify the fibre length to muscle length ratio for the rotator cuff muscles, a parameter that is thought to indicate a muscle's functional capacity for excursion (high ratio) or force production (low ratio) independent of muscle size (Ward et al., 2009). While muscle length is well‐defined for muscles that span the long bones (arm and leg muscles), it was not obvious to us how to define muscle length for muscles like the subscapularis and infraspinatus, with their triangular shapes and broad attachments to the scapula. Finally, our image‐based reconstructions have shown the complexity of muscle architecture and aponeurosis structures in the rotator cuff muscles, but the functional implications were based on simple scalar metrics (e.g., PCSA and mean fascicle lengths). It is plausible that these architectural parameters, and their presumed relationship to muscle function, fail to capture important functional aspects of muscles with complex architectures. Consequently, more advanced 3D modelling approaches may be necessary to explore the functional consequences of muscles with complex architectures in more detail.

It is possible that the muscle architecture measurements reported here are sensitive to tractography stopping criteria (Bolsterlee et al., 2019). However, our previous study showed little sensitivity in the subscapularis (Zhang et al., 2023). In addition, as mentioned in Section 4.1, deterministic fibre tracking algorithms are sensitive to noise and image artefacts in DTI data. Future work could explore alternative DTI‐based techniques such as probabilistic fibre tracking (Behrens et al., 2007; Parker et al., 2003) or population‐averaged modelling of whole‐muscle architecture (Bolsterlee, 2022). These methods could improve architectural measurement accuracy to further advance our understanding of intramuscular, intermuscular, and interindividual variations in muscle architecture.

## AUTHOR CONTRIBUTIONS

**Yilan Zhang**: Conceptualisation, methodology, software, validation, formal analysis, investigation, resources, data curation, visualisation, writing – original draft, writing – review and editing, and project administration. **Robert D. Herbert**: Conceptualisation, methodology, writing – review and editing, project administration, and supervision. **Lynne E. Bilston**: Conceptualisation, validation, writing – review and editing, and supervision. **Bart Bolsterlee**: Conceptualisation, methodology, software, validation, formal analysis, investigation, resources, data curation, visualisation, writing – review and editing, project administration, and supervision.

## CONFLICT OF INTEREST STATEMENT

I hereby state that none of the authors have had any financial or personal relationships with other people or organizations that could inappropriately influence (bias) our work.

## Supporting information


**Table S1.** Muscle architecture and moment arm measurements of all 20 participants included in this study. Values of fascicle length and pennation angle are means ± standard deviations (%CV) over 3000 reconstructed fibre tracts in each muscle.
**TABLE S2.** Mean and median fascicle lengths across 3000 reconstructed fibre tracts in each muscle of all 20 participants included in this study.
**TABLE S3.** Muscle architecture and moment arm measurements from this study and previously published studies. Values are means ± standard deviations across participants.

## Data Availability

The data that support the findings of this study can be made available for research purposes on request to the corresponding author. The data are not publicly available due to ethical restrictions.

## References

[joa14050-bib-0001] Abate, M. , Schiavone, C. , Di Carlo, L. & Salini, V. (2014) Prevalence of and risk factors for asymptomatic rotator cuff tears in postmenopausal women. Menopause, 21, 275–280. 23760436 10.1097/GME.0b013e31829638e3

[joa14050-bib-0002] Aeles, J. , Bolsterlee, B. , Kelp, N.Y. , Dick, T.J.M. & Hug, F. (2022) Regional variation in lateral and medial gastrocnemius muscle fibre lengths obtained from diffusion tensor imaging. Journal of Anatomy, 240, 131–144. 34411299 10.1111/joa.13539 PMC8655206

[joa14050-bib-0003] Andersson, J.L.R. , Skare, S. & Ashburner, J. (2003) How to correct susceptibility distortions in spin‐echo echo‐planar images: application to diffusion tensor imaging. NeuroImage, 20, 870–888. 14568458 10.1016/S1053-8119(03)00336-7

[joa14050-bib-0004] Andersson, J.L.R. & Sotiropoulos, S.N. (2016) An integrated approach to correction for off‐resonance effects and subject movement in diffusion MR imaging. NeuroImage, 125, 1063–1078. 26481672 10.1016/j.neuroimage.2015.10.019 PMC4692656

[joa14050-bib-0005] Bacle, G. , Gregoire, J.‐M. , Patat, F. , Clavert, P. , de Pinieux, G. , Laulan, J. et al. (2017) Anatomy and relations of the infraspinatus and the teres minor muscles: a fresh cadaver dissection study. Surgical and Radiologic Anatomy, 39, 119–126. 27286948 10.1007/s00276-016-1707-9

[joa14050-bib-0006] Basser, P.J. , Pajevic, S. , Pierpaoli, C. , Duda, J. & Aldroubi, A. (2000) In vivo fiber tractography using DT‐MRI data. Magnetic Resonance in Medicine, 44, 625–632. 11025519 10.1002/1522-2594(200010)44:4<625::aid-mrm17>3.0.co;2-o

[joa14050-bib-0007] Bates, K.T. & Falkingham, P.L. (2018) The importance of muscle architecture in biomechanical reconstructions of extinct animals: a case study using *Tyrannosaurus rex* . Journal of Anatomy, 233, 625–635. 30129185 10.1111/joa.12874 PMC6183000

[joa14050-bib-0008] Behrens, T.E.J. , Berg, H.J. , Jbabdi, S. , Rushworth, M.F.S. & Woolrich, M.W. (2007) Probabilistic diffusion tractography with multiple fibre orientations: what can we gain? NeuroImage, 34, 144–155. 17070705 10.1016/j.neuroimage.2006.09.018 PMC7116582

[joa14050-bib-0009] Bird, L. , D'Souza, A. , Ball, I. , Rae, C. , Herbert, R.D. & Bolsterlee, B. (2019) Validity and reliability of measurements of aponeurosis dimensions from magnetic resonance images. Scandinavian Journal of Medicine & Science in Sports, 29, 808–815. 30746780 10.1111/sms.13407

[joa14050-bib-0010] Bolsterlee, B. (2022) A new framework for analysis of three‐dimensional shape and architecture of human skeletal muscles from in vivo imaging data. Journal of Applied Physiology, 132, 712–725. 35050794 10.1152/japplphysiol.00638.2021

[joa14050-bib-0011] Bolsterlee, B. , D'Souza, A. & Herbert, R.D. (2019) Reliability and robustness of muscle architecture measurements obtained using diffusion tensor imaging with anatomically constrained tractography. Journal of Biomechanics, 86, 71–78. 30739766 10.1016/j.jbiomech.2019.01.043

[joa14050-bib-0012] Bolsterlee, B. , Finni, T. , D'Souza, A. , Eguchi, J. , Clarke, E.C. & Herbert, R.D. (2018) Three‐dimensional architecture of the whole human soleus muscle *in vivo* . PeerJ, 6, e4610. 29682414 10.7717/peerj.4610 PMC5910694

[joa14050-bib-0013] Bolsterlee, B. , Gandevia, S.C. & Herbert, R.D. (2016) Effect of transducer orientation on errors in ultrasound image‐based measurements of human medial gastrocnemius muscle fascicle length and pennation. PLoS One, 11, e0157273. 27294280 10.1371/journal.pone.0157273 PMC4905739

[joa14050-bib-0014] Bolsterlee, B. , Vardy, A.N. , van der Helm, F.C.T. & DirkJan Veeger, H.E.J. (2015) The effect of scaling physiological cross‐sectional area on musculoskeletal model predictions. Journal of Biomechanics, 48, 1760–1768. 26050956 10.1016/j.jbiomech.2015.05.005

[joa14050-bib-0015] Broyde, S. , Dempsey, M. , Wang, L. , Cox, P.G. , Fagan, M. & Bates, K.T. (2021) Evolutionary biomechanics: hard tissues and soft evidence? Proceedings of the Royal Society B: Biological Sciences, 288, 20202809. 10.1098/rspb.2020.2809 PMC7935025 33593183

[joa14050-bib-0016] Büchler, P. & Farron, A. (2004) Benefits of an anatomical reconstruction of the humeral head during shoulder arthroplasty: a finite element analysis. Clinical Biomechanics, 19, 16–23. 14659925 10.1016/j.clinbiomech.2003.09.009

[joa14050-bib-0017] Carrasco, D.I. , Lawrence, J. & English, A.W. (1999) Neuromuscular compartments of cat lateral gastrocnemius produce different torques about the ankle joint. Motor Control, 3, 436–446. 10529506 10.1123/mcj.3.4.436

[joa14050-bib-0018] Charles, J. , Kissane, R. , Hoehfurtner, T. & Bates, K.T. (2022) From fibre to function: are we accurately representing muscle architecture and performance? Biological Reviews, 97, 1640–1676. 35388613 10.1111/brv.12856 PMC9540431

[joa14050-bib-0019] Damon, B.M. , Froeling, M. , Buck, A.K.W. , Oudeman, J. , Ding, Z. , Nederveen, A.J. et al. (2017) Skeletal muscle diffusion tensor‐MRI fiber tracking: rationale, data acquisition and analysis methods, applications and future directions: skeletal muscle DT‐MRI fiber tracking. NMR in Biomedicine, 30, e3563. 10.1002/nbm.3563 PMC5136336 27257975

[joa14050-bib-0020] D'Souza, A. , Bolsterlee, B. , Lancaster, A. & Herbert, R.D. (2019) Muscle architecture in children with cerebral palsy and ankle contractures: an investigation using diffusion tensor imaging. Clinical Biomechanics, 68, 205–211. 31255994 10.1016/j.clinbiomech.2019.06.013

[joa14050-bib-0021] Ferrari, F.S. , Governi, S. , Burresi, F. , Vigni, F. & Stefani, P. (2002) Supraspinatus tendon tears: comparison of US and MR arthrography with surgical correlation. European Radiology, 12, 1211–1217. 11976869 10.1007/s00330-001-1183-3

[joa14050-bib-0022] Franchi, M.V. , Raiteri, B.J. , Longo, S. , Sinha, S. , Narici, M.V. & Csapo, R. (2018) Muscle architecture assessment: strengths, shortcomings and new frontiers of in vivo imaging techniques. Ultrasound in Medicine & Biology, 44, 2492–2504. 30185385 10.1016/j.ultrasmedbio.2018.07.010

[joa14050-bib-0023] Gans, C. (1982) Fiber architecture and muscle function. Exercise and Sport Sciences Reviews, 10, 160–207. 6749514

[joa14050-bib-0024] Gates, J.J. , Gilliland, J. , McGarry, M.H. , Park, M.C. , Acevedo, D. , Fitzpatrick, M.J. et al. (2010) Influence of distinct anatomic subregions of the supraspinatus on humeral rotation. Journal of Orthopaedic Research, 28, 12–17. 19621422 10.1002/jor.20947

[joa14050-bib-0025] Gazielly, D.F. , Gleyze, P. & Montagnon, C. (1994) Functional and anatomical results after rotator cuff repair. Clinical Orthopaedics, 304, 43–53. 8020233

[joa14050-bib-0026] Gerber, C. , Fuchs, B. & Hodler, J. (2000) The results of repair of massive tears of the rotator cuff. The Journal of Bone and Joint Surgery, 82, 505–515. 10761941 10.2106/00004623-200004000-00006

[joa14050-bib-0027] Gröning, F. , Jones, M.E.H. , Curtis, N. , Herrel, A. , O'Higgins, P. , Evans, S.E. et al. (2013) The importance of accurate muscle modelling for biomechanical analyses: a case study with a lizard skull. Journal of the Royal Society Interface, 10, 20130216. 23614944 10.1098/rsif.2013.0216 PMC3673157

[joa14050-bib-0028] Herring, S.W. , Grimm, A.F. & Grimm, B.R. (1979) Functional heterogeneity in a multipinnate muscle. The American Journal of Anatomy, 154, 563–575. 433798 10.1002/aja.1001540410

[joa14050-bib-0029] Hess, S.A. (2000) Functional stability of the glenohumeral joint. Manual Therapy, 5, 63–71. 10903581 10.1054/math.2000.0241

[joa14050-bib-0030] Higham, T.E. & Biewener, A.A. (2011) Functional and architectural complexity within and between muscles: regional variation and intermuscular force transmission. Philosophical Transactions of the Royal Society, B: Biological Sciences, 366, 1477–1487. 10.1098/rstb.2010.0359 PMC3130453 21502119

[joa14050-bib-0031] Hik, F. & Ackland, D.C. (2019) The moment arms of the muscles spanning the glenohumeral joint: a systematic review. Journal of Anatomy, 234, 1–15. 30411350 10.1111/joa.12903 PMC6284439

[joa14050-bib-0032] Hughes, R.E. (1996) Force analysis of rotator cuff muscles. Clinical Orthopaedics and Related Research, 330, 75–83. 10.1097/00003086-199609000-00010 8804277

[joa14050-bib-0033] Isensee, F. , Jaeger, P.F. , Kohl, S.A.A. , Petersen, J. & Maier‐Hein, K.H. (2021) nnU‐net: a self‐configuring method for deep learning‐based biomedical image segmentation. Nature Methods, 18, 203–211. 33288961 10.1038/s41592-020-01008-z

[joa14050-bib-0034] Janssen, I. , Heymsfield, S.B. , Wang, Z. & Ross, R. (2000) Skeletal muscle mass and distribution in 468 men and women aged 18–88 yr. Journal of Applied Physiology, 89, 81–88. 10904038 10.1152/jappl.2000.89.1.81

[joa14050-bib-0035] Jenkinson, M. , Bannister, P. , Brady, M. & Smith, S. (2002) Improved optimization for the robust and accurate linear registration and motion correction of brain images. NeuroImage, 17, 825–841. 12377157 10.1016/s1053-8119(02)91132-8

[joa14050-bib-0036] Jenkinson, M. & Smith, S. (2001) A global optimisation method for robust affine registration of brain images. Medical Image Analysis, 5, 143–156. 11516708 10.1016/s1361-8415(01)00036-6

[joa14050-bib-0037] Juul‐Kristensen, B. , Bojsen‐Møller, F. , Finsen, L. , Eriksson, J. , Johansson, G. , Ståhlberg, F. et al. (2000) Muscle sizes and moment arms of rotator cuff muscles determined by magnetic resonance imaging. Cells, Tissues, Organs, 167, 214–222. 10971045 10.1159/000016784

[joa14050-bib-0038] Kälin, P.S. , Crawford, R.J. , Marcon, M. , Manoliu, A. , Bouaicha, S. , Fischer, M.A. et al. (2018) Shoulder muscle volume and fat content in healthy adult volunteers: quantification with DIXON MRI to determine the influence of demographics and handedness. Skeletal Radiology, 47, 1393–1402. 29687149 10.1007/s00256-018-2945-1

[joa14050-bib-0039] Keating, J. , Waterworth, P. , Shaw‐Dunn, J. & Crossan, J. (1993) The relative strengths of the rotator cuff muscles. A cadaver study. Journal of Bone and Joint Surgery. British Volume (London), 75‐B, 137–140. 10.1302/0301-620X.75B1.8421011 8421011

[joa14050-bib-0040] Khandare, S. , Arce, R.A. & Vidt, M.E. (2022) Muscle compensation strategies to maintain glenohumeral joint stability with increased rotator cuff tear severity: a simulation study. Journal of Electromyography and Kinesiology, 62, 102335. 31324511 10.1016/j.jelekin.2019.07.005 PMC13286037

[joa14050-bib-0041] Kim, S. , Bleakney, R. , Boynton, E. , Ravichandiran, K. , Rindlisbacher, T. , McKee, N. et al. (2010) Investigation of the static and dynamic musculotendinous architecture of supraspinatus. Clinical Anatomy, 23, 48–55. 19941361 10.1002/ca.20896

[joa14050-bib-0042] Kim, S.Y. , Bleakney, R.R. , Rindlisbacher, T. , Ravichandiran, K. , Rosser, B.W.C. & Boynton, E. (2013) Musculotendinous architecture of pathological supraspinatus: a pilot in vivo ultrasonography study. Clinical Anatomy, 26, 228–235. 22431385 10.1002/ca.22065

[joa14050-bib-0043] Kim, S.Y. , Boynton, E.L. , Ravichandiran, K. , Fung, L.Y. , Bleakney, R. & Agur, A.M. (2007) Three‐dimensional study of the musculotendinous architecture of supraspinatus and its functional correlations. Clinical Anatomy, 20, 648–655. 17352416 10.1002/ca.20469

[joa14050-bib-0044] Kramer, P.A. , Feuerriegel, E.M. , Lautzenheiser, S.G. & Sylvester, A.D. (2022) Sensitivity of musculoskeletal models to variation in muscle architecture parameters. Evolutionary Human Sciences, 4, e6. 37588892 10.1017/ehs.2022.6 PMC10426084

[joa14050-bib-0045] Kuwahara, Y. , Yuri, T. , Fujii, H. & Kiyoshige, Y. (2017) Functions of the subregions of the infraspinatus during lateral rotation. Surgical and Radiologic Anatomy, 39, 1331–1336. 28600654 10.1007/s00276-017-1886-z

[joa14050-bib-0046] Langenderfer, J. , Jerabek, S.A. , Thangamani, V.B. , Kuhn, J.E. & Hughes, R.E. (2004) Musculoskeletal parameters of muscles crossing the shoulder and elbow and the effect of sarcomere length sample size on estimation of optimal muscle length. Clinical Biomechanics, 19, 664–670. 15288451 10.1016/j.clinbiomech.2004.04.009

[joa14050-bib-0047] Langenderfer, J.E. , Patthanacharoenphon, C. , Carpenter, J.E. & Hughes, R.E. (2006) Variation in external rotation moment arms among subregions of supraspinatus, infraspinatus, and teres minor muscles. Journal of Orthopaedic Research, 24, 1737–1744. 16779813 10.1002/jor.20188 PMC1551907

[joa14050-bib-0048] Lee, D. , Li, Z. , Sohail, Q.Z. , Jackson, K. , Fiume, E. & Agur, A. (2015) A three‐dimensional approach to pennation angle estimation for human skeletal muscle. Computer Methods in Biomechanics and Biomedical Engineering, 18, 1474–1484. 24849037 10.1080/10255842.2014.917294

[joa14050-bib-0049] Lieber, R.L. & Fridén, J. (2000) Functional and clinical significance of skeletal muscle architecture. Muscle & Nerve, 23, 1647–1666. 11054744 10.1002/1097-4598(200011)23:11<1647::aid-mus1>3.0.co;2-m

[joa14050-bib-0050] Lorensen, W.E. & Cline, H.E. (1987) Marching cubes: a high resolution 3D surface construction algorithm. ACM SIGGRAPH Computer Graphics, 21, 163–169.

[joa14050-bib-0051] Mathewson, M.A. , Kwan, A. , Eng, C.M. , Lieber, R.L. & Ward, S.R. (2014) Comparison of rotator cuff muscle architecture between humans and other selected vertebrate species. The Journal of Experimental Biology, 217, 261–273. 24072803 10.1242/jeb.083923 PMC3898624

[joa14050-bib-0052] Meskers, C.G.M. , Van Der Helm, F.C.T. , Rozendaal, L.A. & Rozing, P.M. (1997) In vivo estimation of the glenohumeral joint rotation center from scapular bony landmarks by linear regression. Journal of Biomechanics, 31, 93–96. 10.1016/s0021-9290(97)00101-2 9596544

[joa14050-bib-0053] Miller, A.E.J. , MacDougall, J.D. , Tarnopolsky, M.A. & Sale, D.G. (1993) Gender differences in strength and muscle fiber characteristics. European Journal of Applied Physiology, 66, 254–262. 10.1007/BF00235103 8477683

[joa14050-bib-0054] Motabar, H. & Nimbarte, A.D. (2021) Sex differences in rotator cuff muscles' response to various work‐related factors. IISE Transactions on Occupational Ergonomics and Human Factors, 9, 1–12. 34011247 10.1080/24725838.2021.1931562

[joa14050-bib-0055] Narici, M.V. , Maganaris, C.N. , Reeves, N.D. & Capodaglio, P. (2003) Effect of aging on human muscle architecture. Journal of Applied Physiology, 95, 2229–2234. 12844499 10.1152/japplphysiol.00433.2003

[joa14050-bib-0056] Parker, G.J.M. , Haroon, H.A. & Wheeler‐Kingshott, C.A.M. (2003) A framework for a streamline‐based probabilistic index of connectivity (PICo) using a structural interpretation of MRI diffusion measurements. Journal of Magnetic Resonance Imaging, 18, 242–254. 12884338 10.1002/jmri.10350

[joa14050-bib-0057] Persad, L.S. , Binder‐Markey, B.I. , Shin, A.Y. , Lieber, R.L. & Kaufman, K.R. (2023) Computer models do not accurately predict human muscle passive muscle force and fiber length: evaluating subject‐specific modeling impact on musculoskeletal model predictions. Journal of Biomechanics, 159, 111798. 37713970 10.1016/j.jbiomech.2023.111798

[joa14050-bib-0058] Roh, M.S. , Wang, V.M. , April, E.W. , Pollock, R.G. , Bigliani, L.U. & Flatow, E.L. (2000) Anterior and posterior musculotendinous anatomy of the supraspinatus. Journal of Shoulder and Elbow Surgery, 9, 436–440. 11075329 10.1067/mse.2000.108387

[joa14050-bib-0059] Schenk, P. , Siebert, T. , Hiepe, P. , Güllmar, D. , Reichenbach, J.R. , Wick, C. et al. (2013) Determination of three‐dimensional muscle architectures: validation of the dti ‐based fiber tractography method by manual digitization. Journal of Anatomy, 223, 61–68. 23678961 10.1111/joa.12062 PMC4487763

[joa14050-bib-0060] Sherman, M.A. , Seth, A. & Delp, S.L. (2013) What is a moment arm? Calculating muscle effectiveness in biomechanical models using generalized coordinates. Proceedings of the ASME Design Engineering Technical Conference, 2013. 10.1115/DETC2013-13633 PMC4404026 25905111

[joa14050-bib-0061] Shrout, P.E. & Fleiss, J.L. (1979) Intraclass correlations: uses in assessing rater reliability. Psychological Bulletin, 86, 420–428. 18839484 10.1037//0033-2909.86.2.420

[joa14050-bib-0062] Smith, R.E. , Tournier, J.‐D. , Calamante, F. & Connelly, A. (2012) Anatomically‐constrained tractography: improved diffusion MRI streamlines tractography through effective use of anatomical information. NeuroImage, 62, 1924–1938. 22705374 10.1016/j.neuroimage.2012.06.005

[joa14050-bib-0063] Smith, S.M. , Jenkinson, M. , Woolrich, M.W. , Beckmann, C.F. , Behrens, T.E.J. , Johansen‐Berg, H. et al. (2004) Advances in functional and structural MR image analysis and implementation as FSL. NeuroImage, 23(Suppl 1), S208–S219. 15501092 10.1016/j.neuroimage.2004.07.051

[joa14050-bib-0064] Takahashi, K. , Shiotani, H. , Evangelidis, P.E. , Sado, N. & Kawakami, Y. (2022) Three‐dimensional architecture of human medial gastrocnemius fascicles in vivo: regional variation and its dependence on muscle size. Journal of Anatomy, 241, 1324–1335. 36004517 10.1111/joa.13750 PMC9644967

[joa14050-bib-0065] Tournier, J.‐D. , Smith, R. , Raffelt, D. , Tabbara, R. , Dhollander, T. , Pietsch, M. et al. (2019) MRtrix3: a fast, flexible and open software framework for medical image processing and visualisation. NeuroImage, 202, 116137. 31473352 10.1016/j.neuroimage.2019.116137

[joa14050-bib-0066] Van Hooren, B. , Teratsias, P. & Hodson‐Tole, E.F. (2020) Ultrasound imaging to assess skeletal muscle architecture during movements: a systematic review of methods, reliability, and challenges. Journal of Applied Physiology, 128, 978–999. 32163334 10.1152/japplphysiol.00835.2019

[joa14050-bib-0067] Veraart, J. , Fieremans, E. & Novikov, D.S. (2016) Diffusion MRI noise mapping using random matrix theory: diffusion MRI noise mapping. Magnetic Resonance in Medicine, 76, 1582–1593. 26599599 10.1002/mrm.26059 PMC4879661

[joa14050-bib-0068] Veraart, J. , Novikov, D.S. , Christiaens, D. , Ades‐aron, B. , Sijbers, J. & Fieremans, E. (2016) Denoising of diffusion MRI using random matrix theory. NeuroImage, 142, 394–406. 27523449 10.1016/j.neuroimage.2016.08.016 PMC5159209

[joa14050-bib-0069] Vidt, M.E. , Santago, A.C. , Marsh, A.P. , Hegedus, E.J. , Tuohy, C.J. , Poehling, G.G. et al. (2018) Modeling a rotator cuff tear: individualized shoulder muscle forces influence glenohumeral joint contact force predictions. Clinical Biomechanics, 60, 20–29. 30308434 10.1016/j.clinbiomech.2018.10.004 PMC6252115

[joa14050-bib-0070] Ward, S.R. , Eng, C.M. , Smallwood, L.H. & Lieber, R.L. (2009) Are current measurements of lower extremity muscle architecture accurate? Clinical Orthopaedics, 467, 1074–1082. 10.1007/s11999-008-0594-8 PMC2650051 18972175

[joa14050-bib-0071] Ward, S.R. , Hentzen, E.R. , Smallwood, L.H. , Eastlack, R.K. , Burns, K.A. , Fithian, D.C. et al. (2006) Rotator cuff muscle architecture: implications for glenohumeral stability. Clinical Orthopaedics, 448, 157–163. 10.1097/01.blo.0000194680.94882.d3 16826111

[joa14050-bib-0072] Ward, S.R. , Winters, T.M. & Blemker, S.S. (2010) The architectural design of the gluteal muscle group: implications for movement and rehabilitation. The Journal of Orthopaedic and Sports Physical Therapy, 40, 95–102. 20118527 10.2519/jospt.2010.3302

[joa14050-bib-0073] Wellmann, M. (2016) Diagnostics and treatment of anterosuperior rotator cuff tears. Orthopedics, 45, 130–135. 10.1007/s00132-015-3215-8 26781802

[joa14050-bib-0074] Yushkevich, P.A. , Piven, J. , Hazlett, H.C. , Smith, R.G. , Ho, S. , Gee, J.C. et al. (2006) User‐guided 3D active contour segmentation of anatomical structures: significantly improved efficiency and reliability. NeuroImage, 31, 1116–1128. 16545965 10.1016/j.neuroimage.2006.01.015

[joa14050-bib-0075] Zhang, Y. , Herbert, R.D. , Bilston, L.E. & Bolsterlee, B. (2023) Three‐dimensional architecture of the human subscapularis muscle in vivo. Journal of Biomechanics, 161, 111854. 10.1016/j.jbiomech.2023.111854 39492188

