# Supplementary material for: Three‐dimensional architecture and moment arms of human rotator cuff muscles in vivo: Interindividual, intermuscular, and intramuscular variations
Source: J Anat. 2024 May 1;245(2):258–70. doi: 10.1111/joa.14050 (PMC11259750; doi:10.1111/joa.14050)
Supplement: Supplementary file 1 — Table S1. Muscle architecture and moment arm measurements of all 20 participants included in this study. Values of fascicle length and pennation angle are means ± standard deviations (%CV) over 3000 reconstructed fibre tracts in each muscle. TABLE S2. Mean and median fascicle lengths across 3000 reconstructed fibre tracts in each muscle of all 20 participants included in this study. TABLE S3. Muscle architecture and moment arm measurements from this study and previously published studies. Values are means ± standard deviations across participants. [file JOA-245-258-s001.zip › tables.docx]

# Supplementary material

**Table S1*.*** Muscle architecture and moment arm measurements of all 20 participants included in this study. Values of fascicle length and pennation angle are means ± standard deviations (%CV) over 3000 reconstructed fibre tracts in each muscle.

| Subject | | 1 | 2 | 3 | 4 | 5 | 6 | 7 |
| --- | --- | --- | --- | --- | --- | --- | --- | --- |
| Muscle volume (cm^3^) | SUP | 56 | 34 | 34 | 55 | 51 | 70 | 61 |
|  | SUB | 169 | 90 | 85 | 168 | 140 | 172 | 130 |
|  | INF | 151 | 65 | 70 | 172 | 129 | 157 | 116 |
|  | TER | 32 | 17 | 10 |  | 28 | 42 | 29 |
| Relative muscle volume (%) | SUP | 63 | 57 | 68 | 63 | 67 | 67 | 69 |
|  | SUB | 94 | 53 | 58 | 57 | 66 | 65 | 63 |
|  | INF | 86 | 60 | 51 | 63 | 66 | 62 | 65 |
|  | TER | 44 | 37 | 34 |  | 41 | 41 | 49 |
| PCSA (cm^2^) | SUP | 9 | 6 | 5 | 9 | 8 | 11 | 9 |
|  | SUB | 21 | 17 | 15 | 30 | 21 | 26 | 21 |
|  | INF | 18 | 11 | 14 | 27 | 20 | 25 | 18 |
|  | TER | 5 | 5 | 3 |  | 7 | 10 | 6 |
| Fascicle length (mm) | SUP | 62.6 ± 17.5 (28) | 56.6 ± 15.2 (27) | 67.8 ± 22.4 (33) | 62.9 ± 14.8 (24) | 66.7 ± 14.7 (22) | 66.6 ± 19.9 (30) | 68.5 ± 13.7 (20) |
|  | SUB | 93.9 ± 22.4 (24) | 52.9 ± 16.5 (31) | 58.2 ± 24.4 (42) | 56.7 ± 20.1 (36) | 66.3 ± 34.1 (51) | 65.0 ± 27.7 (43) | 62.9 ± 22.5 (36) |
|  | INF | 86.0 ± 23.9 (28) | 59.7 ± 24.1 (40) | 51.1 ± 20.9 (41) | 62.8 ± 17.2 (27) | 65.6 ± 23.4 (36) | 62.0 ± 22.9 (37) | 64.6 ± 22.2 (34) |
|  | TER | 43.5 ± 10.1 (23) | 37.2 ± 7.8 (21) | 34.4 ± 6.9 (20) |  | 40.8 ± 13.3 (33) | 41.3 ± 8.1 (20) | 49.3 ± 12.8 (26) |
| Pennation angle (°) | SUP | 18 ± 10 (58) | 15 ± 11 (73) | 23 ± 10 (41) | 17 ± 11 (66) | 17 ± 10 (63) | 17 ± 11 (68) | 17 ± 9 (52) |
|  | SUB | 22 ± 12 (53) | 19 ± 10 (52) | 21 ± 11 (52) | 21 ± 11 (55) | 24 ± 12 (52) | 22 ± 12 (57) | 20 ± 11 (54) |
|  | INF | 24 ± 12 (50) | 23 ± 12 (50) | 23 ± 12 (52) | 21 ± 12 (56) | 22 ± 11 (50) | 19 ± 11 (57) | 20 ± 9 (48) |
|  | TER | 24 ± 15 (63) | 24 ± 11 (45) | 20 ± 14 (70) |  | 28 ± 16 (57) | 22 ± 12 (57) | 19 ± 10 (50) |
| Moment arm (mm) | SUP | 25.4 | 24.9 | 22.9 | 23.8 | 25.6 | 24.6 | 19.3 |
|  | SUB | 24.4 | 22.0 | 21.9 | 22.9 | 23.9 | 24.3 | 25.8 |
|  | INF | 25.7 | 19.0 | 22.5 | 22.8 | 25.6 | 25.0 | 25.4 |
|  | TER | 24.5 | 21.9 | 19.1 |  | 24.4 | 23.3 | 23.0 |

| Subject | | 8 | 9 | 10 | 11 | 12 | 13 | 14 |
| --- | --- | --- | --- | --- | --- | --- | --- | --- |
| Muscle volume (cm^3^) | SUP | 50 | 35 | 60 | 48 | 33 | 50 | 37 |
|  | SUB | 196 | 102 | 153 | 143 | 103 | 188 | 92 |
|  | INF | 146 | 96 | 150 | 120 | 68 | 136 | 72 |
|  | TER | 32 |  | 30 | 29 | 20 | 30 | 18 |
| Relative muscle volume (%) | SUP | 46 | 57 | 43 | 64 | 55 | 53 | 54 |
|  | SUB | 56 | 61 | 56 | 63 | 56 | 57 | 56 |
|  | INF | 61 | 59 | 86 | 71 | 64 | 75 | 75 |
|  | TER | 33 |  | 35 | 40 | 47 | 50 | 37 |
| PCSA (cm^2^) | SUP | 11 | 6 | 14 | 7 | 6 | 9 | 7 |
|  | SUB | 35 | 17 | 27 | 23 | 18 | 33 | 17 |
|  | INF | 24 | 16 | 17 | 17 | 11 | 18 | 10 |
|  | TER | 10 |  | 9 | 7 | 4 | 6 | 5 |
| Fascicle length (mm) | SUP | 46.5 ± 8.6 (18) | 56.6 ± 11.3 (20) | 43.4 ± 18.2 (42) | 64.1 ± 13.0 (20) | 54.9 ± 17.5 (32) | 53.3 ± 19.6 (37) | 54.1 ± 18.8 (35) |
|  | SUB | 56.1 ± 22.2 (40) | 60.8 ± 23.0 (38) | 56.4 ± 23.2 (41) | 63.1 ± 25.1 (40) | 55.9 ± 25.2 (45) | 57.1 ± 20.9 (37) | 55.6 ± 20.2 (36) |
|  | INF | 60.8 ± 15.3 (25) | 58.6 ± 21.4 (36) | 86.3 ± 32.4 (38) | 71.1 ± 21.4 (30) | 63.5 ± 16.0 (25) | 75.4 ± 24.3 (32) | 75.0 ± 18.4 (24) |
|  | TER | 33.2 ± 6.5 (20) |  | 34.8 ± 9.5 (27) | 40.2 ± 10.6 (26) | 46.5 ± 8.0 (17) | 50.4 ± 16.7 (33) | 37.3 ± 11.6 (31) |
| Pennation angle (°) | SUP | 21 ± 13 (63) | 14 ± 9 (64) | 23 ± 12 (52) | 16 ± 10 (63) | 16 ± 10 (63) | 13 ± 8 (62) | 16 ± 13 (79) |
|  | SUB | 24 ± 14 (57) | 21 ± 12 (56) | 23 ± 13 (56) | 21 ± 13 (63) | 21 ± 11 (54) | 21 ± 13 (64) | 19 ± 12 (63) |
|  | INF | 18 ± 11 (62) | 23 ± 12 (54) | 18 ± 13 (75) | 17 ± 10 (61) | 21 ± 12 (52) | 21 ± 12 (56) | 18 ± 12 (69) |
|  | TER | 19 ± 13 (66) |  | 22 ± 12 (56) | 21 ± 11 (55) | 19 ± 15 (81) | 25 ± 14 (56) | 26 ± 12 (44) |
| Moment arm (mm) | SUP | 26.0 | 23.3 | 24.7 | 21.8 | 24.7 | 24.8 | 25.9 |
|  | SUB | 25.8 | 24.7 | 23.7 | 24.4 | 23.2 | 21.7 | 23.6 |
|  | INF | 24.7 | 25.9 | 25.8 | 24.6 | 19.8 | 23.4 | 25.0 |
|  | TER | 21.9 |  | 23.5 | 20.9 | 23.5 | 24.9 | 22.8 |

| Subject | | 15 | 16 | 17 | 18 | 19 | 20 |
| --- | --- | --- | --- | --- | --- | --- | --- |
| Muscle volume (cm^3^) | SUP | 30 | 31 | 36 | 67 | 51 | 48 |
|  | SUB | 100 | 88 | 106 | 214 | 203 | 109 |
|  | INF | 77 | 64 | 96 | 158 | 137 | 114 |
|  | TER | 22 | 17 |  | 32 | 52 |  |
| Relative muscle volume (%) | SUP | 66 | 61 | 54 | 57 | 69 | 63 |
|  | SUB | 63 | 64 | 65 | 72 | 55 | 57 |
|  | INF | 68 | 60 | 64 | 98 | 68 | 66 |
|  | TER | 46 | 43 |  | 47 | 46 |  |
| PCSA (cm^2^) | SUP | 5 | 5 | 7 | 12 | 7 | 8 |
|  | SUB | 16 | 14 | 16 | 30 | 37 | 19 |
|  | INF | 11 | 11 | 15 | 16 | 20 | 17 |
|  | TER | 5 | 4 |  | 7 | 11 |  |
| Fascicle length (mm) | SUP | 66.2 ± 14.5 (22) | 61.0 ± 14.2 (23) | 53.7 ± 9.5 (18) | 56.8 ± 16.2 (29) | 69.5 ± 14.6 (21) | 63.4 ± 14.5 (23) |
|  | SUB | 62.6 ± 25.3 (40) | 64.4 ± 23.0 (36) | 64.7 ± 23.8 (37) | 72.1 ± 30.3 (42) | 55.2 ± 22.1 (40) | 56.7 ± 37.3 (66) |
|  | INF | 67.8 ± 15.6 (23) | 59.9 ± 18.3 (31) | 63.6 ± 18.6 (29) | 98.3 ± 34.7 (35) | 67.5 ± 21.2 (31) | 65.6 ± 23.3 (35) |
|  | TER | 45.8 ± 15.6 (34) | 43.1 ± 13.3 (31) |  | 47.5 ± 13.7 (29) | 45.6 ± 13.0 (29) |  |
| Pennation angle (°) | SUP | 16 ± 8 (51) | 15 ± 10 (67) | 17 ± 10 (55) | 15 ± 9 (60) | 16 ± 13 (78) | 16 ± 9 (58) |
|  | SUB | 20 ± 11 (55) | 18 ± 11 (57) | 19 ± 11 (61) | 18 ± 11 (57) | 23 ± 12 (50) | 24 ± 11 (44) |
|  | INF | 19 ± 10 (54) | 18 ± 10 (56) | 18 ± 10 (56) | 20 ± 12 (61) | 20 ± 12 (60) | 20 ± 12 (58) |
|  | TER | 23 ± 12 (51) | 23 ± 14 (61) |  | 18 ± 11 (60) | 22 ± 13 (58) |  |
| Moment arm (mm) | SUP | 24.7 | 24.3 | 25.9 | 24.3 | 22.1 | 24.5 |
|  | SUB | 20.6 | 21.4 | 20.6 | 25.7 | 23.7 | 22.6 |
|  | INF | 25.3 | 24.5 | 24.7 | 23.8 | 25.8 | 25.2 |
|  | TER | 23.3 | 22.5 |  | 22.2 | 24.0 |  |

CV = coefficient of variation; PCSA = physiological cross-sectional area; SUP = supraspinatus; SUB = subscapularis; INF = infraspinatus; TER = teres minor.

**Table S2.** Mean and median fascicle lengths across 3000 reconstructed fibre tracts in each muscle of all 20 participants included in this study.

| Subject | SUP | | SUB | | INF | | TER | |
| --- | --- | --- | --- | --- | --- | --- | --- | --- |
|  | mean | median | mean | median | mean | median | mean | median |
| 1 | 62.6 | 63.3 | 93.9 | 98.1 | 86.0 | 85.0 | 43.5 | 41.2 |
| 2 | 56.6 | 54.2 | 52.9 | 50.6 | 59.7 | 55.3 | 37.2 | 35.0 |
| 3 | 67.8 | 71.3 | 58.2 | 52.7 | 51.1 | 46.7 | 34.4 | 33.9 |
| 4 | 62.9 | 60.5 | 56.7 | 55.2 | 62.8 | 61.0 |  | |
| 5 | 66.7 | 65.7 | 66.3 | 55.6 | 65.6 | 69.3 | 40.8 | 35.6 |
| 6 | 66.6 | 64.5 | 65.0 | 63.3 | 62.0 | 64.6 | 41.3 | 40.6 |
| 7 | 68.5 | 67.6 | 62.9 | 61.4 | 64.6 | 66.8 | 49.3 | 45.0 |
| 8 | 46.5 | 46.2 | 56.1 | 53.6 | 60.8 | 58.4 | 33.2 | 32.0 |
| 9 | 56.6 | 56.0 | 60.8 | 56.6 | 58.6 | 54.7 |  | |
| 10 | 43.4 | 35.9 | 56.4 | 51.9 | 86.3 | 82.9 | 34.8 | 32.6 |
| 11 | 64.1 | 63.2 | 63.1 | 62.9 | 71.1 | 68.5 | 40.2 | 40.5 |
| 12 | 54.9 | 57.3 | 55.9 | 48.5 | 63.5 | 61.6 | 46.5 | 47.6 |
| 13 | 53.3 | 49.4 | 57.1 | 54.0 | 75.4 | 76.5 | 50.4 | 46.4 |
| 14 | 54.1 | 54.1 | 55.6 | 51.0 | 75.0 | 72.0 | 37.3 | 32.6 |
| 15 | 66.2 | 65.5 | 62.6 | 56.9 | 67.8 | 66.9 | 45.8 | 39.9 |
| 16 | 61.0 | 63.2 | 64.4 | 62.9 | 59.9 | 57.8 | 43.1 | 39.3 |
| 17 | 53.7 | 52.5 | 64.7 | 64.7 | 63.6 | 63.6 |  | |
| 18 | 56.8 | 59.1 | 72.1 | 66.1 | 98.3 | 106.5 | 47.5 | 46.2 |
| 19 | 69.5 | 69.3 | 55.2 | 50.9 | 67.5 | 67.6 | 45.6 | 44.8 |
| 20 | 63.4 | 63.9 | 56.7 | 41.3 | 65.6 | 63.4 |  | |
| **Average** | 59.7 | 59.1 | 61.8 | 57.9 | 68.3 | 67.5 | 41.9 | 39.6 |
| **Standard deviation** | 7.2 | 8.4 | 8.7 | 11.1 | 10.9 | 12.7 | 5.2 | 5.2 |

SUP = supraspinatus; SUB = subscapularis; INF = infraspinatus; TER = teres minor.

**Table S3*.*** Muscle architecture and moment arm measurements from this study and previously published studies. Values are means ± standard deviations across participants.

| Studies | | Muscle volume (cm^3^) | PCSA (cm^2^) | Fascicle length (mm)^d^ | Pennation angle (°) | Moment arm (mm) |
| --- | --- | --- | --- | --- | --- | --- |
| SUP | This study^a^ | 47 ± 12 | 8 ± 2 | 59.8 ± 7.2 | 17 ± 3 | 24.2 ± 1.6 |
|  | Ward et al. (2006)^b^ | 31 ± 12 | 7 ± 2 | 45.0 ± 10.1 | 5 ± 1 | - |
|  | Mathewson et al. (2014)^b^ | 40 ± 13 | 8 ± 3 | 56.5 ± 10 | 8 ± 5 | - |
|  | Juul-Kristensen et al. (2000)^b, c^ | 30 ± 12 | 7 ± 3 | 47.0 ± 11.0 | 11 ± 8 | - |
|  | Juul-Kristensen et al. (2000)^a, c^ | 48 ± 8 | - | - | - | 24.0 ± 1.0 |
| SUB | This study^a^ | 138 ± 42 | 23 ± 7 | 61.8 ± 8.7 | 21 ± 2 | 23.3 ± 1.6 |
|  | Ward et al. (2006)^b^ | 92 ± 33 | 16 ± 5 | 60.0 ± 14.9 | 0 ± 0 | - |
|  | Mathewson et al. (2014)^b^ | 117 ± 42 | 19 ± 7 | 63.6 ± 12.7 | 13 ± 9 | - |
|  | Juul-Kristensen et al. (2000)^b, c^ | 90 ± 20 | 19 ± 7 | 50.0 ± 14.0 | 13 ± 8 | - |
|  | Juul-Kristensen et al. (2000)^a, c^ | 154 ± 22 | - | - | - | 23.0 ± 1.0 |
| INF | This study^a^ | 113 ± 36 | 17 ± 5 | 69.7 ± 11.7 | 20 ± 2 | 24.2 ± 2.0 |
|  | Ward et al. (2006)^b^ | 70 ± 21 | 11 ± 3 | 65.7 ± 10.4 | 1 ± 0 | - |
|  | Mathewson et al. (2014)^b^ | 93 ± 29 | 13 ± 4 | 76.0 ± 14.1 | 12 ± 10 | - |
|  | Juul-Kristensen et al. (2000)^b, c^ | 84 ± 9 | 14 ± 3 | 65.0 ± 12.0 | 12 ± 5 | - |
|  | Juul-Kristensen et al. (2000)^a, c^ | 125 ± 16 | - | - | - | 23.0 ± 2.0 |
| TER | This study^a^ | 28 ± 10 | 6 ± 2 | 41.9 ± 5.2 | 22 ± 3 | 22.9 ± 1.4 |
|  | Ward et al. (2006)^b^ | 19 ± 6 | 3 ± 1 | 60.9 ± 11.1 | 1 ± 1 | - |
|  | Mathewson et al. (2014)^b^ | 23 ± 9 | 4 ± 2 | 72.5 ± 14.0 | 7 ± 3 | - |
|  | Juul-Kristensen et al. (2000)^b, c^ | - | - | - | - | - |
|  | Juul-Kristensen et al. (2000)^a, c^ | - | - | - | - | - |

PCSA = physiological cross-sectional area; SUP = supraspinatus; SUB = subscapularis; INF = infraspinatus; TER = teres minor.

^a^ Measurements were obtained from in vivo MRI scans from healthy living humans.

^b^ Measurements were obtained from cadaveric dissections.

^c^ The infraspinatus and teres minor muscles were grouped together and measured as one muscle (designated infraspinatus).

^d^ This study and the cadaveric study by Juul-Kristensen et al. (2000) reported measured fascicle lengths. Cadaveric studies by Ward et al. (2006) and Mathewson et al. (2014) reported normalized fascicle lengths that have been normalized to optimal sarcomere lengths.
